# Insights into tumor vaccines for elderly individuals in the context of immunosenescence

**DOI:** 10.3389/fimmu.2025.1660874

**Published:** 2025-10-08

**Authors:** Chenglong Li, Zhujun Chen, Changyu Zhu, Min Chen, Jinqi Li, Lei Zhong, Yingying Hou

**Affiliations:** ^1^ Department of Pharmacy, Personalized Drug Research and Therapy Key Laboratory of Sichuan Province, Sichuan Provincial People’s Hospital, School of Medicine, University of Electronic Science and Technology of China, Chengdu, China; ^2^ Department of Pharmacy, Sichuan Clinical Medical Research Center for Neurological Diseases, Deyang Key Laboratory of Tumor Molecular Research, Deyang People’s Hospital, Affiliated Hospital of Chengdu University of Traditional Chinese Medicine, Deyang, China

**Keywords:** cancer, immunosenescence, elderly individuals, vaccine efficacy, anti-tumor immunity

## Abstract

The global burden of cancer is increasing tremendously, particularly among individuals aged 60 years and older, and has emerged as a critical public health concern. Cancer vaccine-induced immunity can recognize and eliminate tumor cells with high specificity and low toxicity. Nevertheless, immunosenescence increases the risk and severity of cancers in elderly individuals while impairing vaccine-induced immunity. Furthermore, much oncology research has predominantly focused on adults, often neglecting the potential contributions of aging individuals to tumor progression. Elucidating the interactions between the immunosenescent microenvironment and tumorigenesis can inspire the development of more effective cancer vaccines tailored to the characteristics of elderly individuals, thereby alleviating the global cancer burden. In this review, we analyze how the immunosenescent microenvironment impacts tumor development and summarize existing strategies aimed at enhancing cancer vaccine efficacy, drawing inspiration from insights into immunosenescence. We believe that this review will inspire efforts toward creating individualized cancer vaccines for the elderly.

## Graphical Abstract

Emerging strategies aimed at enhancing the efficacy of cancer vaccines in elderly individuals include inhibiting inflammatory signals, promoting antigen presentation, mitigating T-cell immunosenescence, eliminating senescent cells, utilizing immunomodulatory adjuvants, and employing senescent tumor cell-derived vaccines. (Created with Adobe illustrator)

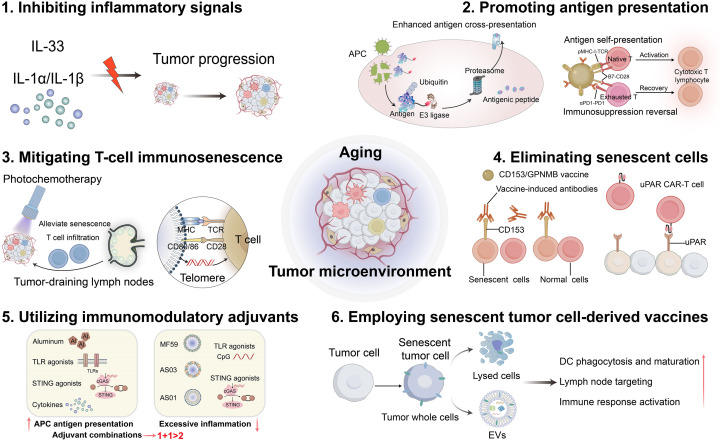

## 1 Introduction

Globally, the proportion of older adults is increasing rapidly; this trend is expected to remain prevalent in demographic changes (1). Age-related diseases are increasingly being recognized as a pressing public health issue, presenting pivotal challenges to healthcare systems globally. Notably, data indicates that malignant tumors have become the second leading cause of death worldwide (2). As the global population ages, both the burden of cancer and mortality rates are expected to rise steadily due to population aging (3). It has long been established that aging is closely associated with the development of tumors, especially in the older population, where cancer incidence tends to increase (4). Moreover, the median age at which cancer is diagnosed is 66 years (5). Consequently, devising effective strategies for tumor prevention and treatment has significant scientific value and societal importance for promoting healthy aging.

Vaccination is an important approach for mitigating age-related diseases by eliciting antigen-specific immunity (6). However, the effectiveness of vaccinations often declines in older adults due to immunosenescence. Immunosenescence refers to a decline in both the quantity and functionality of immune cells, leading to compromised immunity (7). As the immune function wanes, the immune system’s ability to monitor tumor cells decreases, allowing tumors to proliferate and metastasize. A comprehensive understanding of the impact of immunosenescence on tumor vaccines is essential for the development of efficient and personalized cancer vaccines tailored to the elderly.

Cancer vaccines consist of tumor-associated antigens (TAAs) in combination with immunostimulatory adjuvants, designed to target immune cells and stimulate the production of tumor-specific T cell immunity for targeted elimination of tumor cells (8). According to their use, tumor vaccines can be categorized into two main types: prophylactic and therapeutic vaccines. Despite the encouraging outcomes observed in preclinical studies, cancer vaccines have consistently underperformed in clinical trials. At present, only two prophylactic tumor vaccines are available: the human papillomavirus (HPV) vaccine, for the prevention of preventing cervical cancer, and the hepatitis B virus (HBV) vaccine, intended for liver cancer prevention. Three products have been approved by the United States Food and Drug Administration: Sipuleucel-T (Provenge^®^) vaccine for the treatment of prostate cancer, Bacillus Calmette-Gusamrin for bladder cancer, and Talimogene laherparepvec^®^ for locally advanced and metastatic melanoma (9). To date, no other therapeutic tumor vaccine has been approved, which illustrates the challenges associated with their development. The primary obstacles that diminish the efficacy of therapeutic cancer vaccines include tumor heterogeneity, immunosuppressive tumor microenvironment (TME), immune escape mechanisms, inadequate T cell responses, and suboptimal vaccine formulation (10, 11). Cancer and aging share certain hallmarks related to genomic instability and epigenetic alterations (12). Neoantigen-based cancer vaccines are promising therapeutic targets. Interestingly, senescent cells also express novel antigens owing to their genetic and epigenetic modifications (6). A more thorough understanding of the commonalities and interactions between aging processes and tumors may pave the way for breakthroughs in the development of more effective therapeutic tumor vaccines.

In this review, we analyzed the effects of the aging microenvironment on the TME and its impact on the immune potency of tumor vaccines. We will pay special attention to studying how aging affects different components of the TME, including dendritic cells (DCs), macrophages, B cells, T cells and tumor cells. Additionally, emerging strategies aimed at enhancing the efficacy of cancer vaccines in elderly individuals include inhibiting inflammatory signals, promoting antigen presentation, mitigating T-cell immunosenescence, eliminating senescent cells, utilizing immunomodulatory adjuvants, and employing senescent tumor cell-derived vaccines (Graphical abstract). Our goal was to stimulate interest in individualized vaccine approaches while providing innovative insights to improve cancer vaccination strategies tailored to older adults.

## 2 Immune cell senescence in TME

Tumor vaccines are designed to stimulate the production of tumor-specific T cell immunity, and offer new hope for cancer therapy (13). The basic principles required for successful tumor vaccination include delivery of substantial amounts of appropriate antigens to antigen-presenting cells (APCs), optimal activation of APCs, production of a robust CD4^+^ T cell and cytotoxic T lymphocyte (CTL) response, infiltration into the TME, and persistence and maintenance of anti-tumor immunity (14). However, numerous factors impede tumor vaccine potency, including: (1) inhibitory immune cells, such as regulatory T cells (Tregs), myeloid-derived suppressor cells, tumor-associated macrophages, and cancer-associated fibroblasts (14); (2) inhibitory factors, such as transforming growth factor-beta, interleukin-10 (IL-10), and indoleamine 2,3-dioxygenase; (3) abnormal angiogenesis leading to inefficient blood perfusion that obstructs immune cell infiltration (15); and (4) an immunosuppressive microenvironment characterized by acidic pH, reduced nutrients and oxygen, elevated reactive oxygen species and enzyme expression (16, 17). Addressing these immunosuppressive factors within the TME has emerged as a critical scientific challenge to overcome the application bottleneck in therapeutic tumor vaccines. However, the immunosenescence observed in the elderly further exacerbates the immunosuppressive TME, of the tumor and thus promotes tumor progression. Understanding the age-related changes in immune cell populations within the TME may help in the development of effective tumor vaccines.

**Figure 1:**
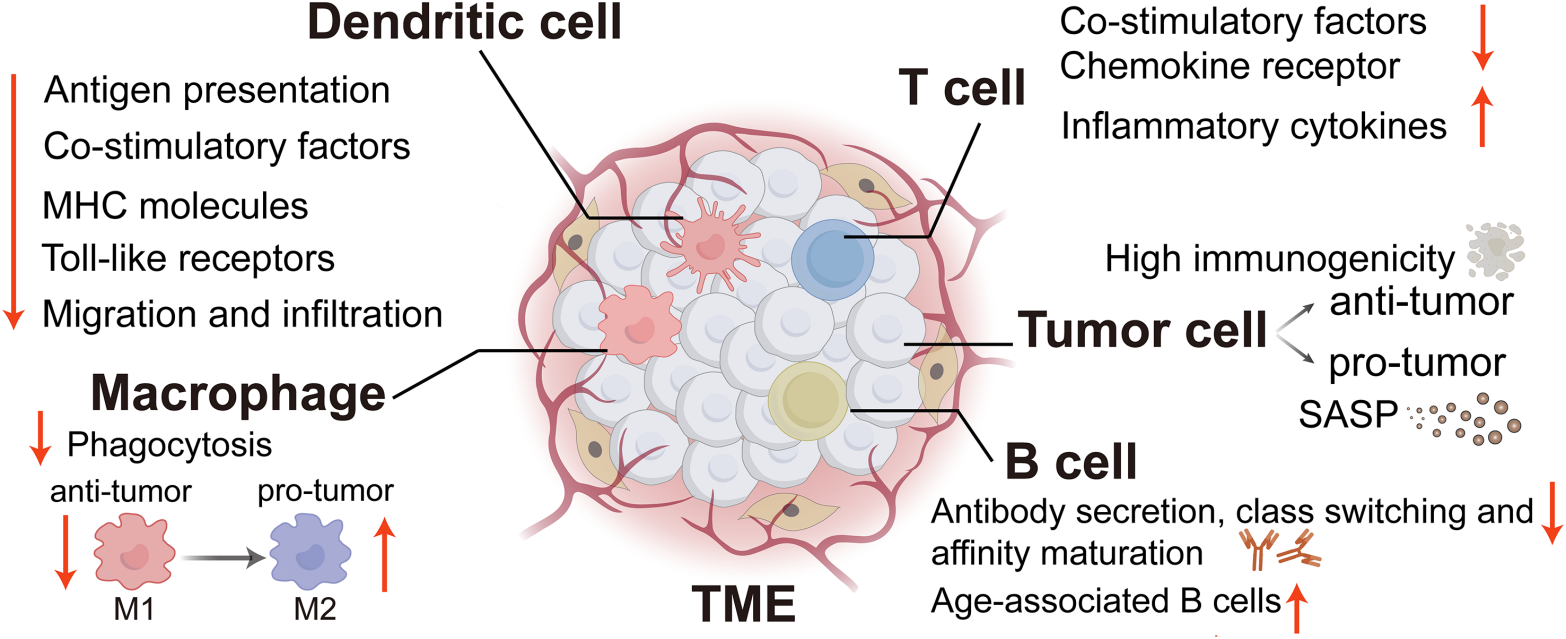
The immune alterations linked to cellular senescence within the TME. TME, tumor microenvironment; SASP, Senescence-associated secretory phenotype; MHC, major histocompatibility complex. (Created with Adobe illustrator).

Aberrant activation of various signaling pathways, including but not limited to NF-κB, mTOR, JAK-STAT, cGAS (cyclic GMP-AMP synthase)-STING (stimulator of interferon genes), AMPK (AMP activated protein kinase), melatonin, and sirtuin pathways, forms a regulatory network to modulate immunosenescence and immune functions (18). Meanwhile, senescence-associated secretory phenotypes (SASPs) also play a pivotal role in mediating the pro-tumor effects associated with senescent cells. SASP represents the secretion of pro-inflammatory cytokines, chemokines, growth factors and other substances that accelerate inflammatory senescence and tumor progression by immune-related tumor cells (19). In the following analyzes, we will systematically investigate the immunological changes associated with cellular senescence within the TME (**Figure 1**) and elucidate the molecular mechanisms by which these changes influence tumor development.

### 2.1 DCs

Impaired DC function associated with aging is one factor linked to diminished anti-tumor immunity (20). DC senescence compromises tumor antigen cross-presentation, as well as DC migration, ultimately leading to a reduction in the host’s anti-tumor immunity (21). Additionally, senescent DCs exacerbate paracrine immunosenescence through the secretion of inflammatory extracellular vesicles (EVs), which induce dysfunction in CD4^+^ T cells and further weaken anti-tumor effects (22).

However, a study found that under specific conditions, DCs can enter a “superactivated” state characterized by heightened activity in antigen presentation, cytokine secretion, chemokine production, co-stimulatory molecule expression, and migratory capacity (23). Moreover, hyperactive DCs are marked by enhanced IL-1β secretion and enhanced migratory ability toward lymph nodes. If this superactivated state can be replicated in elderly patients with cancer, it may serve as an effective strategy for combating cancer. A vaccine adjuvant known as 1-palmitoyl-2-glutaryl phosphatidylcholine is capable of correcting migration deficiencies in aged DCs (24). Vaccines that incorporate tumor antigens along with this adjuvant have been shown to correct DC migratory defects, thereby enhancing anti-tumor immune responses in older mice. Si et al. demonstrated that inhibition of PD-L1 and/or STAT3 signaling in DC blocks γδ Treg-induced DC senescence and restores their effector functions, thereby improving the effects of tumor vaccines and immunotherapy (25). These studies offer new potential strategies for preventing age-related dysfunction of innate immune cells within the TME.

### 2.2 Macrophages

Macrophages along with DCs and B cells, constitute another class of professional APCs (26). With potent phagocytic abilities, macrophages engulf and eliminate parasites, foreign bacteria, and abnormal or senescent cells from the body. Beyond their clearance functions, macrophages are integral to regulating inflammation, promoting wound healing, preserving immune homeostasis, and surveilling abnormal cell populations (27). As macrophages age, several functional alterations occur: compromised autophagy due to decreased expression of autophagy-related gene 5 (ATG5); diminished pathogen phagocytosis; reduced antigen presentation from lower major histocompatibility complex (MHC)-II molecules, co-stimulatory factors, and toll-like receptor (TLR) expression; and impaired migration, infiltration, and recruitment abilities (28).

In the TME, macrophages can be classified as M1-like (anti-tumor properties) and M2-like (promote tumor proliferation and activation) subsets (29). Cellular senescence influences metabolic disorders along with immunophenotypic changes in macrophages while facilitating inhibitory remodeling within the TME that supports tumor progression (30, 31). During this process, macrophages are increasingly transformed into pro-inflammatory phenotypes, which contribute to the secretion of SASP factors. These SASP factors further modify the phenotypic characteristics of macrophages (32). Aging macrophages may show an anti-tumor effect during the early stages of disease; however, as tumor development progresses, they can promote tumor progression and metastasis (28).

Research has demonstrated that the absence of the IL-4-STAT6 signaling pathway can impair DNA repair mechanisms in macrophages (33). It can also activate the cGAS-STING pathway and promote macrophage senescence. Deng et al. revealed that a key metabolic characteristic of the aging TME is the increased production of adenosine (34). Senescent tumor cells secrete IL-6 and promote the expression of CD73 in macrophages, which facilitates the conversion of ADP and AMP into adenosine in the TME, thereby promoting tumor immunosuppression (34, 35). Targeted inhibition of macrophage CD73 in the aging TME can trigger anti-tumor immune responses while inhibiting tumor. Hou et al. have shown that senescent bone marrow macrophages (BMM) release EVs containing specific microRNAs, thereby propagating aging signals to multiple tissues (36). They also identified peroxisome proliferator-activated receptor α as a target of these microRNAs within aged BMM-EVs, which regulates downstream effects related to cellular senescence.

### 2.3 T cells

T cells possess cytotoxic functions and cytokine secretion capabilities, making them crucial effector cells in anti-tumor immunity. Additionally, owing to their high proliferation rates, T cells are more susceptible to aging than other immune cell types (37). Senescent T cells exhibit downregulation of co-stimulatory receptors, such as CD27, CD28, and chemokine receptor CCR7; conversely, they upregulate inflammatory and immunomodulatory cytokines and chemokines, including TNF-α, IL-1β, and IL-6 (7). Due to mitochondrial dysfunction, senescent T cells demonstrate heightened activation of the cGAS–STING, PI3K-AKT-mTOR and MAPK signaling pathways (38). Loss of tumor antigen-specific CD8^+^ T cells is a major feature that accelerates tumor growth and leads to immunotherapy resistance in older mice (39). However, at later stages following the induction of aging, inhibitory properties may be acquired by PD-L1 or other unidentified signals among T-cells (40). Notably, Chen et al.’s analysis of single-cell sequencing data from infiltrating CD8^+^ T lymphocytes derived from mice revealed a subset characterized by PD-1^+^Tox^+^IL-7R^+^CD8^+^, known as age-related dysfunctional tumor-infiltrating lymphocytes (T-Tad) (41). The proportion of T-Tad cells in tumors derived from aged mice was significantly higher than that observed in young mice. Notably, subsequent studies have demonstrated that treatment with a CD40 agonist significantly augments the populations of cDC1s and CD8^+^ T cells within tumors from elderly mice, while also restoring cDC1 antigen-presenting capabilities and alleviating age-related dysfunction of CD8^+^ T cells. Thus, CD40 agonists could rescue age-related deficits in CD8^+^ T-cell immunity and tumor control.

The deficiency of CD4^+^ T cells in the elderly can also significantly affect immunity. CD4^+^ T cells were traditionally regarded as primarily “helper” cells, responsible for secreting chemokines that recruit and sustain an effective cellular immune response (42). Recent investigations have shown that cytotoxic CD4^+^ T cells also possess direct anti-tumor activity against several common tumors (43). Further functional studies have indicated that they primarily exert their effects by directly targeting tumors through MHC II molecules (44). The exhausted, cytotoxic, and activated regulatory T cell subsets within CD4^+^ T cells are infrequently observed in young mice but progressively accumulate with advancing age (45). Notably surprising are the extreme pro-inflammatory and anti-inflammatory phenotypes exhibited by cytotoxic CD4 T cells and activated regulatory cells, respectively. A comprehensive understanding of the specific roles and regulatory mechanisms that govern CD4^+^ T cell function in tumor immunotherapy is crucial for designing next-generation tumor vaccines.

Future investigations should further examine phenotypic changes across various T cell subsets distinctly affected by aging. This could help tumor vaccines tailored to different T-cell subpopulations for refined regulation. Chen et al. reported that with advancing age, the γδT cell pool within peripheral lymph nodes becomes entirely skewed toward the γδ17 lineage while concurrently witnessing a substantial reduction in γδ1T cell numbers (46). These findings underscore the pivotal role of the γδT cell population residing within peripheral lymph nodes in modulating the balance between pro- and anti-tumor immunity. Thus, an inclination toward pro-tumorigenic CD17 lineages during aging may serve as a pivotal factor contributing to age-related increases in tumor incidence. Moreover, γδT cells present significant advantages over αβT cells. They are not restricted by MHC or histocompatibility leukocyte antigen presentation (47), rendering them suitable for allotherapy.

### 2.4 B cells

In numerous solid tumors, B cells represent the second most abundant lymphocyte population, constituting 40-50% of intratumoral lymphocytes, 25% of the overall tumor cell population, and 30% of the cellular composition within tumor-draining lymph nodes (TDLNs) (48). B cells can exert various anti-tumor immune effects by presenting tumor antigens to activate both CD4^+^ T helper cells and CD8^+^ cytotoxic T lymphocytes. They secrete diverse effector cytokines while expressing granzyme B, as well as ligands such as tumor necrosis factor-related apoptosis-inducing ligands and Fas ligands, which are capable of directly lysing tumor cells (48, 49). Recent investigations suggest that B cells may enhance anti-tumor CD8^+^ T cell responses through IL-27 secretion (50). While research has predominantly focused on DC-based immunotherapy strategies, approaches centered on B cells also show considerable promise. B cells exposed to CD40 or TLR9 agonists showed significantly enhanced T-cell activation (51).

The functions of antibody secretion, class switching, and affinity maturation in senescent B cells are decreased (37). In addition, due to the down-regulation of CD40 expression, the activation of senescent B cells is reduced (18). Age-associated B cells (ABCs) have attracted increasing attention in the past decade. These cells display a distinct surface phenotype and transcriptional profile, relying on signaling from TLR7 or TLR9 in conjunction with Th1 cytokines for their formation and activation (52). Research conducted by Castro et al. examined the accumulation of ABCs in murine models and their correlation with lymphoma development (53). The findings revealed that as mice aged, both the accumulation of ABCs and alterations within the aging microenvironment synergistically contributed to the promotion of lymphoma development.

### 2.5 Tumor cells

While most research on aging has focused on non-cancerous cells, it is important to note that cancer cells also undergo aging. The SASP is a common characteristic of senescent cells. Some anti-tumor interventions can induce senescence in cancer cells. This is achieved by triggering genotoxic stress, overactivation of mitotic signaling, or oxidative stress, which leads to stable cell cycle arrest and SASP induction (54, 55). Aging cancer cells can inhibit neighboring cancer cells and recruit immune cells, thereby further suppressing tumor growth (55). Senescent tumor cells hold significant potential for application in tumor immunotherapy because of their high immunogenicity and activation of APCs, as well as enhanced anti-tumor immunity mediated by DCs and CD8 T lymphocytes (56, 57). This potential can be summarized in two key aspects. First, aging cancer cells remain viable for longer than dead cancer cells. They are capable of stimulating the immune system for an extended period. Second, these aging cancer cells have lost their ability to divide and proliferate, so they do not regenerate into tumors. However, senescent tumor cells may also exhibit immunosuppressive SASP factors and promote tumorigenesis (58). Various SASP components contribute to stemness in cancers as well as proliferation, migration, invasion, and metastasis, thereby increasing the malignant potential of cancer cells (59). Understanding and leveraging the interplay between immunosenescence in tumor cells and tumors will aid in improving the rational design of tumor vaccines.

## 3 Design strategies for cancer vaccines for elderly individuals

The efficacy of vaccines in elderly individuals is significantly diminished. However, several strategies have been proposed to enhance vaccine-induced immunity. As summarized in our previously published review, approaches to improve vaccination effectiveness among older adults include increasing the antigen dose, developing multivalent antigen vaccines, incorporating suitable adjuvants, inhibiting chronic inflammation, and counteracting immunosenescence (37). Nevertheless, owing to the complexities associated with the aging TME, simply increasing antigen doses or preparing polyvalent antigen vaccines may not be optimal for tumor vaccine design. We outlined strategies for enhancing tumor vaccines inspired by the aging TME. These strategies include inhibiting inflammatory signals, promoting antigen presentation, mitigating T-cell immunosenescence, eliminating senescent cells, utilizing immunomodulatory adjuvants, and employing senescent tumor cell-derived vaccines.

### 3.1 Inhibiting inflammation signals

Inflammation is a common feature of aging and cancer. Currently, anti-inflammatory therapies have been shown to have the potential to slow both the aging process and tumor progression (37, 60). Several anti-inflammatory drugs currently under development have demonstrated significant anti-tumor effects. For instance, canakinumab, a recombinant human antibody targeting IL-1β, was initially designed for the treatment of cardiovascular disease but was serendipitously found to reduce lung cancer mortality in clinical trials (60). IL-33 serves as a crucial initiator of chronic inflammation; by binding to tumorigenesis suppressor 2, it fosters the establishment of a chronic inflammatory environment within stressed and inflamed tissues. Researchers discovered that the commonly used cholesterol-lowering medication pitavastatin can inhibit the progression of chronic inflammation into cancer by blocking IL-33 expression (61). These findings provide compelling evidence to support the application of anti-inflammatory therapies in cancer prevention. Park et al. established that immune system aging is a critical determinant of lung cancer outcome (62). Specifically, myeloid progenitor-like cell-derived IL-1α is a key driver of the aging-enhanced acute myelopoietic response. Blocking this signaling pathway using anti-IL-1α/IL-1β antibodies during the early stages of tumor development can reverse the pro-cancer effects attributed to an aging immune system.

Consequently, we speculated that targeting pro-inflammatory cytokines through vaccination may serve as a promising strategy for alleviating both aging and cancer progression. However, these strategies still necessitate long-term safety data from the elderly population for validation. Significantly, IL-33 plays a crucial role in the vaccine-induced Th2-type immune response, while IL-1β can function as an adjuvant to enhance cellular immune responses (63, 64). Excessive suppression of inflammation may adversely impact the immune response elicited by the vaccine. Therefore, it is essential to balance the advantages and disadvantages of such therapies while determining the optimal level of anti-inflammatory intervention. Concurrently, attention must be paid to potential side effects and individual variations in response to ensure both safety and efficacy.

### 3.2 Promoting antigen presentation in APCs

Cytotoxic T cell activation against tumor cells usually necessitates antigen cross-presentation by APCs. Recent research has introduced various strategies, including the use of cell-penetrating peptides, the proton sponge effect, membrane destabilization, and photochemical internalization release techniques, to enhance the endolysosomal escape of tumor antigens, thereby improving antigen cross-presentation (65). However, the efficiency of antigen presentation is limited. In addition, both senescent DCs and macrophages exhibit reduced antigen-presenting capabilities (19). Consequently, promoting effective antigen presentation in APCs remains a significant challenge for the development of highly effective anti-tumor vaccines for elderly individuals.

**Figure 2:**
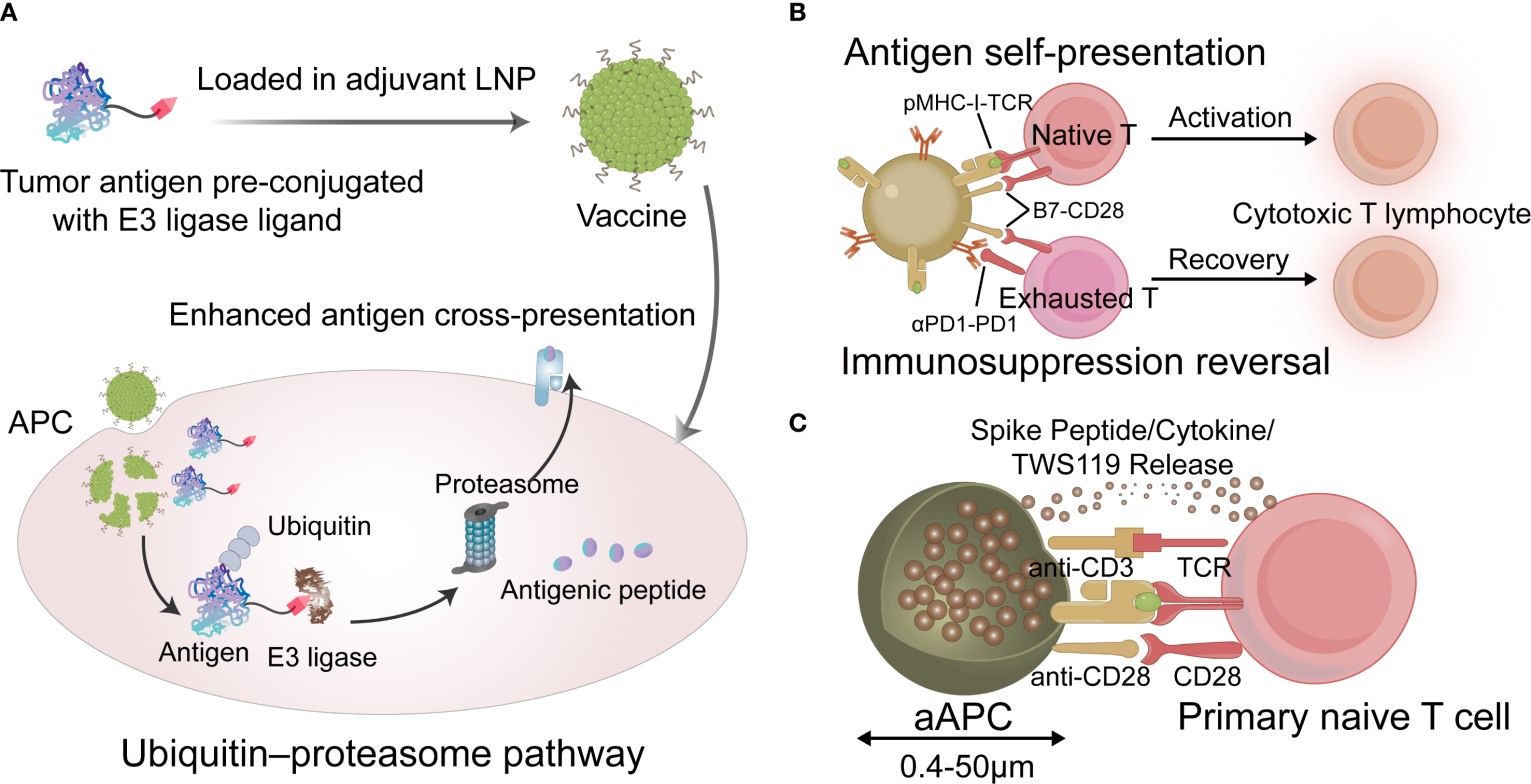
**(A)** The tumor vaccine that leverages targeted antigen degradation to enhance antigen processing. **(B)** Schematic describing vaccine for antigen self-presentation and immunosuppression blockade. **(C)** Schematic diagram of the aAPCs vaccine. LNP, lipid nanoparticles; APC, antigen-presenting cell; TCR, T cell receptor; aAPC, artificial APC. (Created with Adobe illustrator).

The key step in antigen processing is the proteolysis of tumor antigens mediated by the ubiquitin–proteasome pathway(UPP). Zhao et al. reported a tumor vaccine that leverages targeted antigen degradation to significantly enhance antigen processing (66). This vaccine comprises a tumor antigen pre-coupled with an E3 ligand and lipid nanoparticles that target the lymph nodes. The ligand recruits the E3 ubiquitin ligase von Hippel-Lindau protein, which directs tumor antigens to the UPP, thereby enhancing cross-presentation and potentiating anti-tumor T cell responses. To further enhance antigen presentation efficiency, Liu et al. utilized engineered dendritic cell membranes as natural immune activation signal transduction carriers to regulate co-stimulatory molecule expression on dendritic cell surfaces and construct self-presenting nanovaccines (67). Unlike traditional vaccine delivery methods, this approach enables the direct delivery of epitopes to T cells, bypassing the involvement of endogenous antigen-presenting cells. This mode of antigen presentation significantly enhances immune activation efficiency and demonstrates strong efficacy in treating solid tumors. Additionally, artificial APCs(aAPCs) can mimic the functions of natural APCs by directly activating T cells; these should be decorated with peptide-MHC complexes along with co-stimulatory molecules to provide both primary and secondary signals necessary for T cell activation (49). An engineered aAPC was developed to induce CD8 ^+^ T memory stem cells(TMSCs) (68). The results demonstrated that a single injection of the vaccine elicited long-lasting CD8^+^ TMSCs in both young and aged mice, generating humoral immunity that was either stronger than or comparable to that induced by soluble vaccines. Emerging strategies for improving antigen presentation, particularly cross-presentation, are shown in **Figure 2**.

### 3.3 Mitigating T cell immunosenescence

An increasing body of research has demonstrated that T cell aging is a primary contributor to disease in elderly individuals (69). T cells are crucial as effector cells in vaccine responses, mediating cellular immunity and targeting infected or tumorigenic cells for destruction (70, 71). However, the capacity of T cells to recognize and eliminate pathogenic microorganisms or tumor cells significantly diminishes with advancing age (38). Notably, immunosenescence observed in CD8^+^ T cells within TDLNs promotes immune evasion by tumor cells and facilitates cancer progression (72). Senescent T cells present within the TME have emerged as novel targets for cancer immunotherapy (73). Nevertheless, reports on nanovaccines designed to reverse senescent T-cell phenotypes remain scarce. Sui et al. found that the anti-tumor efficacy of sialic acid-modified liposome-mediated non-photochemotherapy declined in 16-month-old mice, and tumors recurred after scabbing (74). Interestingly, sialic acid-modified liposome-mediated photochemotherapy increased infiltration of CD8^+^ T cell and neutrophil into tumors from TDLNs in immunosuppressed mice. This treatment also inhibited metastasis and recurrence of tumors. Because the solid stress exerted by the tumor hinders T cells from infiltrating the tumor, the quantity of T cells in the lymph nodes typically remains unchanged (75). Thymus atrophy in aging mice results in a reduction of T cells in the TDLN and the ability of tumor invasion (38). Phototherapy can reduce tumor cell populations, alter solid pressure within the TDLN, and enhance the infiltration of neutrophils and CD8 T cells into the tumor (74). Additionally, Liu et al. reported that aggregation-induced emission photosensitizer-loaded nanoparticles can activate T cells and alleviate their senescence to reshape the tumor immune microenvironment (76). These studies demonstrated that nanovaccines can reverse T cell senescence; however, a comprehensive understanding of the mechanisms responsible for inhibiting T-cell immunosenescence necessitates additional research. Notably, there are currently no reports on specifically designed targeted nanovaccines aimed at senescent T cells. Moreover, an intriguing discovery revealed that telomeres can be transferred from APCs to T cells via EVs (77). This transfer may prevent T cells from aging and enhance long-term immune memory. Taken together, these observations suggest that telomere transfer could represent a significant breakthrough in mitigating T cell immunosenescence through telomere extension.

### 3.4 Eliminating senescent cells

**Figure 3:**
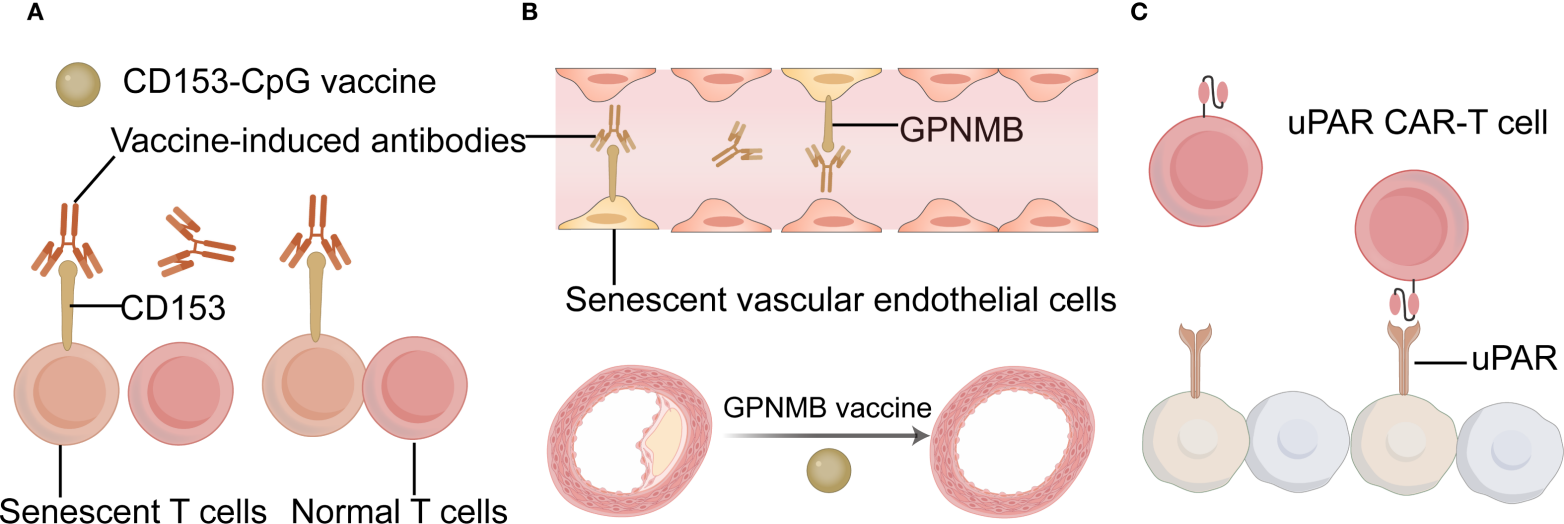
The examples for eliminating senescent cells. **(A)** Schematic diagram of the CD153-CpG vaccine. **(B)** Schematic diagram of the GPNMB vaccine. **(C)** Schematic diagram of the uPAR CAR T cell. CAR chimeric antigen receptor; GPNMB glycoprotein nonmetastatic melanoma protein B; uPAR urokinase-type plasminogen activator receptor; CAR T chimeric antigen receptor T-cell Immunotherapy. (Created with Adobe illustrator).

Cell senescence plays a key role in inducing immunotherapy resistance. Eliminating senescent cells can restore immune homeostasis in the TME, thereby enhancing the effects of immunotherapy (78). The pathological accumulation of senescent cells generates inflammation, causing chronic tissue damage and leading to age-related diseases (79). A new strategy to eliminate senescent cells may inhibit the development of age-related diseases. Tumor vaccines that target molecules highly expressed in senescent tumor cells but not in non-senescent cells can help remove senescent tumor cells (6). Recently, proof-of-concept studies have been conducted on vaccines targeting senescent cells with elevated levels of CD153 or glycoprotein nonmetastatic melanoma protein B (GPNMB) (80, 81). CD153 is a member of the TNF superfamily and is expressed in induced T-cells, monocytes, and macrophages. Yoshida et al. reported that a vaccine based on CD153 epitope-coupled CpG as an adjuvant could induce antigen-specific antibodies and reduce the population of senescent T cells (81). Although GPNMB can act as an aging cell antigen, deleting GPNMB in normal cells may cause adverse effects due to off-target impacts. Therefore, it is important to consider the timing of GPNMB vaccination. Vaccination should be restricted to periods when senescent cells begin to accumulate to avoid compromising normal cell function. Genetically modified immune cells represent another method for targeting senescent cells. By analyzing the transcriptome of senescent cells, proteins that are widely expressed and specifically anchored to these cells can be identified. Amor et al. selected *PLAUR*, a gene encoding the urokinase-type plasminogen activator receptor (uPAR), as the target gene for chimeric antigen receptor (CAR) T cells (82). uPAR CAR-T cells also eliminated UPAR-positive senescent cells and prolonged the survival of senescent mice. Targeted elimination of senescent cells expressing high levels of uPAR may concurrently inhibit tumor angiogenesis. Therefore, the elimination of senescent tumor cells using targeted uPAR vaccine approaches has therapeutic potential. CAR-T therapy falls under the regulations of gene therapy and must adhere to Good Manufacturing Practice standards, undergoing rigorous toxicity assessments and long-term safety evaluations. Furthermore, common side effects associated with CAR-T therapy include cytokine release syndrome and immune effector cell-associated neurotoxicity syndrome, both of which warrant careful consideration. The examples for eliminating senescent cells were summarized in **Figure 3**.

However, in some cases, the depletion of senescent cells may alter vaccine-induced immune responses. Cobanoglu et al. utilized a preclinical melanoma model to evaluate the anti-tumor potential of senolysis prior to vaccination (83). The results showed that the Bcl-2 family inhibitor ABT-263 eliminated the vaccine’s ability to inhibit melanoma growth in older animals. Therefore, more detailed research is needed to determine whether removing senescent cells is an appropriate strategy.

### 3.5 Utilizing immunomodulatory adjuvants

Most tumor antigens are less immunogenic and require adjuvants to help trigger innate immunity and induce a more robust Th1 response, while mitigating tumor-associated immunosuppression (84, 85). Immune adjuvants may be the most promising strategy for boosting immunity in elderly patients. However, achieving maximal anti-tumor immune response and clinical efficacy depends on carefully optimizing adjuvant selection for each vaccine formulation and patient (5). The adjuvants used in tumor vaccines include aluminum adjuvants, Toll-like receptor (TLR) agonists, STING agonists, and cytokines (5, 9). Recently, adjuvant development has focused on agonists of natural immune system receptors, particularly TLR. These adjuvants stimulate the maturation of innate immune cells, promote antigen presentation, and stimulate T cells by activating the pattern recognition receptor (PRR) signaling pathway, thereby improving the effectiveness of cancer vaccines (86). One of the most common adjuvants used in cancer vaccines is the TLR9 agonist CpG-ODN, which is moderately effective when injected intratumorally in glioblastoma patients (87). CpG-ODN also enhances cellular and/or humoral immune responses and promotes Th1 responses in old mice (88). STING serves as an intracellular PRR for cyclic dinucleotides, which are implicated in the immune response to both bacterial infections and intracellular DNA derived from foreign entities or altered self-sources (89). Studies have found that STING activation can boost anti-tumor efficiency in the TME by activating the antigen-presenting capacity of DCs and macrophages and expanding the infiltration of tumor-reactive CD8^+^T cells (90, 91).

**Figure 4:**
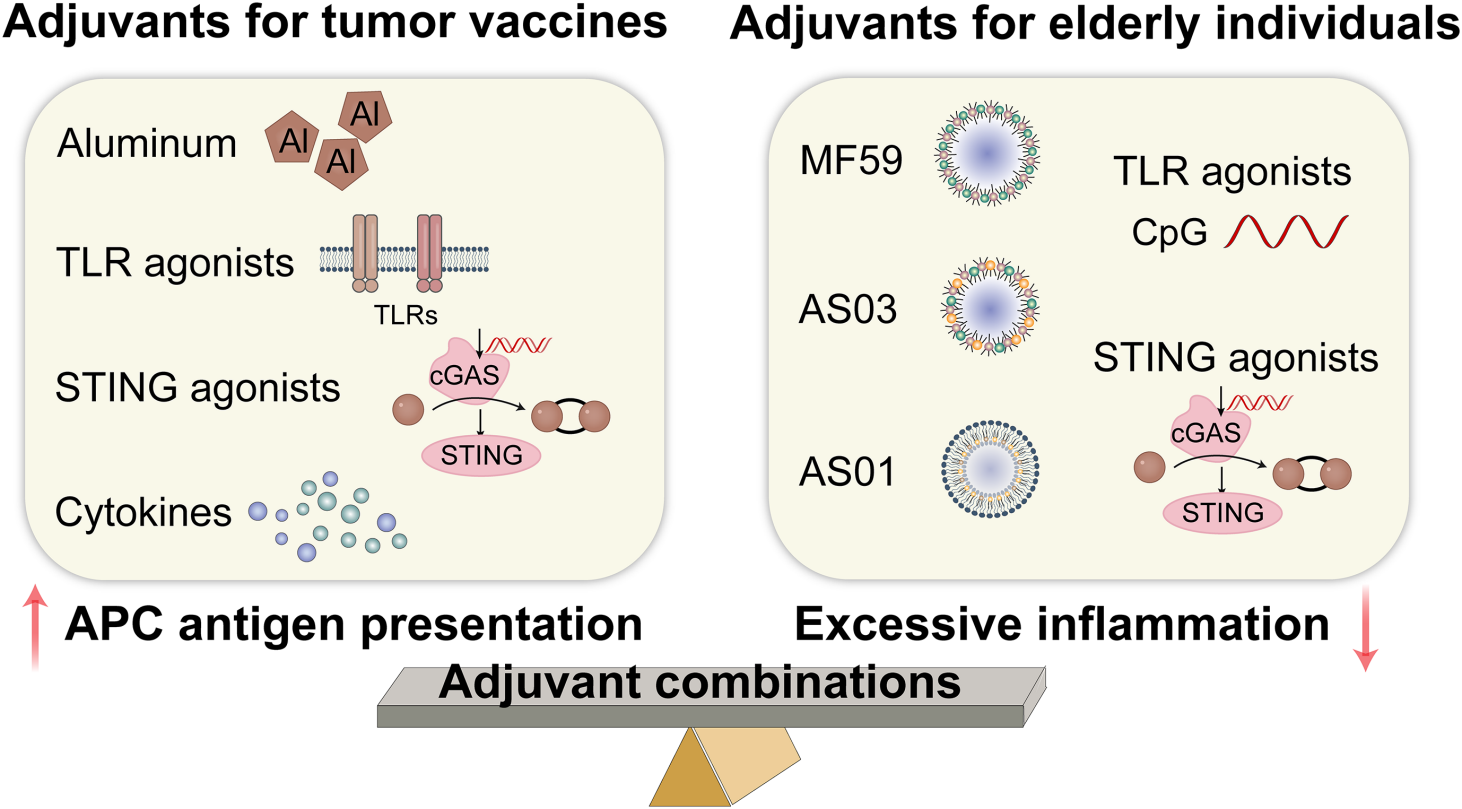
Adjuvants for tumor vaccines in elderly individuals. TLR, toll-like receptor; STING, stimulator of interferon gene; cGAS, cyclic GMP-AMP synthase. (Created with Adobe illustrator).

Ideal adjuvants should not only facilitate innate and adaptive immune responses but also produce durable protective memory (92, 93). Because CD8^+^ T cells play a key role in eradicating tumor cells (94), adjuvants that can promote tumor antigen cross-presentation in DCs and trigger cellular responses are certainly more effective. Given the chronic inflammation present within the TME and in elderly individuals, it is essential that adjuvants enhance APC antigen presentation function without provoking excessive inflammatory responses in these cells (37). The novel nanoadjuvants (polyanhydride nanoparticles and pentablock copolymer micelles) and cyclic dinucleotides (a STING agonist) demonstrated a modest induction of cytokine secretion with a minimal inflammatory profile to overcome immunosenescence in elderly individuals (95). A review showed that MF59, AS03, AS01, and CpG oligodeoxynucleotide (CpG-ODN) adjuvants are effective in older adults (96). Another alternative is to use adjuvant combinations, which can coordinate their advantages to induce more comprehensive immune responses. The AS01B adjuvant used in the recombinant shingles vaccine Shingrix demonstrates a protective efficacy of 89.8% among individuals over 70 years old by activating TLR4 (monophosphoryl lipid A) and enhancing antigen cross-presentation (QS-21) (97). This adjuvant combination also exhibits favorable safety and efficacy profiles when administered to elderly patients with acute myeloid leukemia (98). Consequently, the fundamental mechanism underlying the adjuvant combination strategy is aimed at targeting critical nodes of immune senescence while synergistically modulating the immune microenvironment. The selection of adjuvants for tumor vaccines in elderly individuals is shown in **Figure 4**.

### 3.6 Employing senescent tumor cell-derived vaccines

**Figure 5:**
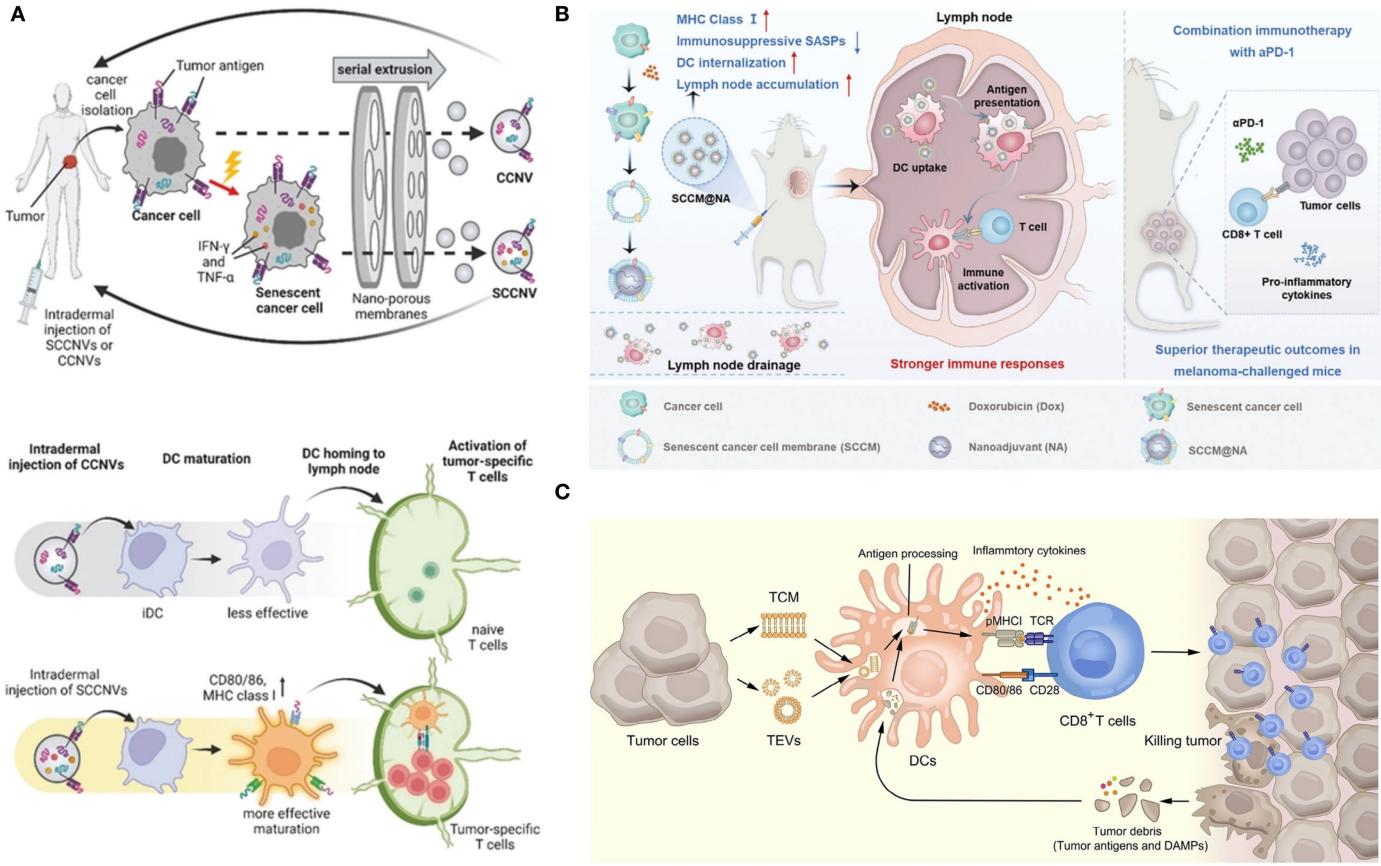
Senescent tumor cell-derived vaccines. **(A)** Description of SCCNV preparation and vaccination and the mode of action of vaccines (99). SCCNV, senescent cancer cell-derived nanovesicle; CCNV, cancer cell-derived nanovesicle; iDC, immature dendritic cell; mDC, mature dendritic cell. **(B)** Schematic illustration of the biomimetic nanovaccines for facilitating DC phagocytosis, promoting lymph node targeting and activating immune responses (100). **(C)** The mechanism of tumor elimination mediated by tumor cell membrane and tumor-derived extracellular vesicles (101). **(A–C)** were reproduced under the terms of the Creative Commons CC-BY 4.0 license.

As mentioned above, senescent tumor cells are double-edged swords with complex modulations. Currently, more research is focused on using the high immunogenicity of senescent tumor cells to design personalized tumor vaccines. Senescent tumor whole cells, lysed cells, and EVs can all serve as sources of tumor antigens; the vaccines developed from these components are referred to as senescent tumor cell-derived vaccines. Hong et al. prepared senescent cancer cell-derived nanovesicles that provided specific tumor antigens and enhanced anti-tumor immunity without the addition of adjuvants (99). During the induced aging process *in vitro*, nanovaccines expressed aging-associated IFN-γ and TNF-α. These endogenous cytokines can be used as adjuvants to enhance immunogenicity and to improve safety. Yang et al. reported a nanovaccine based on the membrane of senescent cancer cells that facilitated the efficacy of tumor immunotherapy (100). Senescent cancer cells were obtained by incubation with a low dose of doxorubicin for 5 days. Integrating senescent cancer cell membranes and nanoadjuvants to construct biomimetic nanovaccines is more effective than using living senescent cancer cells in facilitating DC phagocytosis, promoting lymph node targeting, and activating the immune response. Compared with biomimetic nanovaccines derived from immunogenic cell death-induced tumor cells, senescent cancer cell-based nanovaccines promoted DC maturation. When combined with αPD-1, it produced a significant anti-tumor immune response, improved efficacy, and reduced side effects in mice challenged with melanoma. The emerging strategies for utilizing senescent tumor cell-derived vaccines are illustrated in **Figure 5**.

Tumor cell membranes and tumor-derived EVs are an interesting source of tumor vaccine antigens as well as integrated vectors for vaccine adjuvants and other therapeutic agents (101). High biocompatibility and anti-tumor immune stimulation abilities are incomparable advantages. The immune effect can be further enhanced through genetic engineering, surface modification, hybridization of other cell membranes (e.g., APC membranes and bacterial outer membrane vesicles), and the addition of adjuvants (101). However, the application of vaccines derived from senescent tumor cells in the elderly encounters several challenges. Whole-cell vaccines demand stringent control over cell culture conditions. The heterogeneity of senescent tumor cells may lead to differences in antigen profiles between batches. Key issues hindering clinical translation include low yields of cell lysates and EVs, variability in batch-to-batch production, and unstable expression of surface molecules (101). Additionally, ethical considerations must be addressed: ensuring informed consent and respecting patient autonomy are paramount. Given the complexity and novelty associated with vaccines derived from senescent tumor cells, it is essential that patients fully comprehend the scientific principles underlying their treatment as well as potential risks and uncertainties involved; thus, respecting their autonomous decisions is critical.

## 4 Conclusions and perspectives

As the elderly population continues to grow, we anticipate a substantial increase in both cancer incidence and mortality rates. In this review, we analyze how aging influences immune cells within TME and their subsequent effects on immune efficacy related to tumor vaccines. Additionally, we summarize emerging strategies aimed at enhancing the effectiveness of cancer vaccines in older populations, including inhibiting inflammatory signals, promoting antigen presentation, mitigating T cell immunosenescence, eliminating senescent cells, utilizing immunomodulatory adjuvants, and employing senescent tumor cell-derived vaccines.

Cancer vaccination holds promise for tumor treatment and metastasis prevention; however, one critical aspect often overlooked in clinical trials is the influence of age on cancer immunotherapy outcomes. Real-world research data on CIMAvax-EGF (a lung cancer vaccine) indicate that the median overall survival for patients under 65 years old is 16.7 months, whereas for those over 65 years old, it is 12.2 months (102). This suggests that non-small cell lung cancer patients under 65 years of age benefit from the treatment. In three clinical trials (Mel43, Mel44, Mel48), patients with resected stage IIB-IV melanoma were vaccinated with a combination of 12 melanoma-associated peptides restricted by class I MHC molecules (103). The cumulative immune response was higher in patients under 64 years of age compared to older individuals. Further analysis of clinical tumor samples are necessary to generate research ideas and directions for fundamental scientific inquiry. Gui et al. evaluated the characteristics of the TME in 813 patients with prostate cancer and developed the Aging Microenvironment Index (AMI) (104). Specific analysis of immune cell types according to AMI levels showed that patients with low AMI exhibited higher levels of resting memory CD4^+^ T cells and M2 macrophages. In contrast, patients with high AMI levels demonstrated elevated levels of activated NK cells, follicular helper T cells, and plasma cells. This approach seeks new tumor vaccine targets by analyzing samples from older patients, thereby helping to translate these findings into practice. Future studies will continue to closely integrate clinical and scientific research to establish a virtuous cycle. To address impaired vaccine responses attributed to immunosenescence, pretreatment strategies may be employed to enhance vaccine efficacy. For instance, administering GM-CSF with adjuvant properties prior to vaccination could facilitate the generation of a greater number of functionally active DCs (105). Given the increasing aging population and the complexities associated with clinical oversight and regulatory approval processes, it may be advantageous to shorten approval timelines. Additionally, identifying effective immune response indicators—such as CD8^+^ CD28^+^ T cells—could serve as a foundation for expedited approvals (106). Finally, long-term follow-up is essential to monitor potential unidentified adverse events and assess the sustained efficacy of tumor vaccines.

Furthermore, additional research is warranted to identify immunosenescence biomarkers across different tumor types in patients. These biomarkers not only represent important approaches for early screening, differential diagnosis, evaluation of therapeutic effects, and assessment of prognosis in tumors but also serve as valuable tools for personalized vaccine design (107). The circulating SASP can be detected in the serum of aged mice and humans and may serve as a biomarker for senescence, thereby enhancing our understanding of immunosenescence and its implications for cancer vaccines targeting the elderly (108). A SEA-CAT study (NCT04113122) focuses on investigating the long-term effects of cisplatin-based chemotherapy on cellular senescence in testicular cancer survivors by utilizing SASP biomarkers. A novel senescence identification tool integrates gene characteristics associated with cell-cycle arrest and senescence pathways to detect senescent cells within tumors *in vivo (* 109). This tool has the potential to reveal the heterogeneity among different clusters of senescent cells, along with their respective SASP profiles and survival mechanisms. Saavedra et al. identified that the proportions of CD8^+^ CD28^-^ T cells, CD4^+^ T cell counts, and the CD4/CD8 ratio may act as predictive biomarkers for CIMAvax-EGF cancer vaccine efficacy in non-small cell lung cancer patients (110). Furthermore, a combined assessment of multiple biomarkers is expected to enhance accuracy and effectiveness in patient stratification during clinical trials.

Tumor heterogeneity is a prevalent and critical characteristic in the processes of tumor initiation and progression. This variability often complicates the development of tumor vaccines, as it becomes challenging to encompass all tumor-specific antigens, resulting in an incomplete immune response (10). Additionally, the diminished uptake and presentation efficiency of tumor antigens by APCs in the elderly population may further exacerbate this issue (37). Consequently, both tumor heterogeneity and immunosenescence collaboratively facilitate tumor advancement, presenting significant challenges for vaccine design. We believe that addressing tumor heterogeneity and immunosenescence through a multi-epitope vaccine strategy could prove more effective; however, further research is necessary to validate this approach.

Inefficient antigen delivery and the absence of effective adjuvants to counteract immunosuppression within the TME present significant challenges for tumor vaccine development (111). Antigen delivery can be optimized by refining various forms of antigen delivery systems; additionally, modifications to the shape, particle size, charge, and surface characteristics of the vaccine may prove beneficial (49). When selecting adjuvants for personalized vaccines, it is imperative to consider various influencing factors, such as antigen type, formulation, and delivery system (9). Given the unique features of the TME and immunosenescent microenvironments, exploring novel combinations of multiple adjuvants could potentially elicit more comprehensive immune responses. However, the immune microenvironment exhibit dynamic changes, and the underlying molecular mechanisms are intricate (112). Research that emphasizes multi-target, multi-mechanism, and hierarchical collaborative vectors may more effectively address the dynamic alterations associated with immunosenescence and TME. In the future, it will be essential to leverage artificial intelligence tools to comprehensively analyze and explore its regulatory principles (113) as well as to optimize intervention strategies for tumors. In the future, utilizing artificial intelligence tools will be crucial for analyzing regulatory principles and optimizing tumor intervention strategies. Integrating multidisciplinary methods could advance individualized cancer treatment for elderly patients. For example, big data analysis can help stratify tumor heterogeneity, and gene editing can regulate the activity and stability of specific immune cells in aging bodies.
